# Traditional Chinese Exercise for Neurodegenerative Diseases: A Bibliometric and Visualized Analysis With Future Directions

**DOI:** 10.3389/fnagi.2022.932924

**Published:** 2022-06-27

**Authors:** Buchan Jiang, Chengyao Feng, Huiling Hu, Daniel George, Tianlong Huang, Zhihong Li

**Affiliations:** ^1^Department of Orthopedics, The Second Xiangya Hospital of Central South University, Changsha, China; ^2^Hunan Key Laboratory of Tumor Models and Individualized Medicine, Changsha, China; ^3^Department of Rehabilitation Medicine, The Second Xiangya Hospital of Central South University, Changsha, China; ^4^Department of Orthopedic Surgery, The Queen Elizabeth Hospital, The University of Adelaide, Adelaide, SA, Australia

**Keywords:** Traditional Chinese Exercises, neurodegenerative diseases, bibliometric analysis, visualization, scientometric analysis, Tai Chi, Parkinson’s disease

## Abstract

**Background:**

Traditional Chinese Exercise (TCE) has expanded out of China into the world and is frequently used in the prevention and treatment of many diseases. Although many studies have explored the ability of TCE as an intervention for neurodegenerative diseases, there are limited articles summarizing the research. The aim of this study was to investigate current research trends around TCE for neurodegenerative diseases and predict future directions for such research.

**Materials and Methods:**

Data was collected from the Web of Science Core Collection (WoSCC). All articles and reviews on TCE in relation to neurodegenerative diseases were retrieved. The data analysis was performed using the CiteSpace (5.8R3) software, and the results were displayed in network maps.

**Results:**

The search identified 220 publications between 1999 and 2021. The United States was the most productive country (*n* = 89), followed by China (*n* = 68). The United States had the greatest centrality, indicating its tremendous global influence and close collaborations with other countries. Fuzhong Li from the Oregon Research Institute, United States, was not only the most prolific author (*n* = 5), but also the most co-cited author (*n* = 120). The Shanghai University of Sport contributed to the most publications (*n* = 12). *PLOS ONE* was the most published journal, while *Movement Disorders* was the most cited journal. Tai Chi, Parkinson’s disease, quality of life, balance, and older adult were the most high-frequency keywords, while Alzheimer’s disease had the highest centrality.

**Conclusion:**

The number of publications on TCE related to neurodegenerative diseases has shown major growth in the past decade. However, there is a need for research institutions to strengthen cooperation between countries and institutions. Tai Chi, Parkinson’s disease (PD), Alzheimer’s disease, older adults and falls reduction have been the recent research focus. It is anticipated that in the future, PD will continue to be a central focus with the effects of Baduanjin, Wuqinxi, and Yijinjing requiring further research.

## Introduction

Neurodegenerative disorders are characterized by the progressive degeneration or loss of neuronal structure and function in the central nervous system. Common neurodegenerative diseases include Alzheimer’s disease (AD), Parkinson’s disease (PD), Huntington’s disease, and motor neurone disease. As the world’s aging population is on the rise, the incidence of neurodegenerative diseases is also growing, with about 50 million individuals affected worldwide (Ehrenberg et al., 2020). Many neurodegenerative diseases still lack effective treatments today, which can ultimately result in the physical and functional disabilities of the patients (Fari et al., 2021). It is of great value to explore potential treatments for neurodegenerative diseases, including complementary and alternative therapies such as traditional Chinese medicine and exercise intervention.

Traditional Chinese Exercise (TCE), which originated in China about 3,000 years ago, is increasingly recognized as an integral part of non-pharmacological intervention (Wang et al., 2015; Li et al., 2020). TCEs, including Tai Chi, Qigong, Baduanjin, Yijinjing, and Wuqinxi, are low-medium-intensity exercises that have a strong theoretical basis of traditional Chinese medicine. Characterized by the inter-coordination of body movement, breathing, and meditation, TCEs are mainly in the form of self-cultivation, sometimes with simple tools such as swords and sticks (Song et al., 2020). Therefore, it has very low requirements for equipment and is not limited by location or facility. Growing evidence has demonstrated that TCE is beneficial for improving both physical and mental health (Wang et al., 2015; Guo et al., 2018; Li W. et al., 2021). Under the idea that exercise is medicine (EIM), TCE has been frequently used in medical treatment and prevention of various diseases in Eastern and Western countries (Song et al., 2020). There is also an increasing number of studies reporting that TCE has been used for treating neurodegenerative disease with positive results. For example, a recent randomized controlled experiment reported that Qigong could improve cognitive function in patients with neurodegenerative diseases (Jin et al., 2020). A recent literature review of complementary therapies in PD suggested that TCEs are equally effective at improving motor symptoms of PD compared to other exercise regimens (Deuel and Seeberger, 2020). However, few studies have comprehensively collected global data related to the application of TCEs for neurodegenerative diseases.

Since the work of Garfield in 1987, bibliometric analysis has become a popular quantitative method to analyze the quality and scholarly impact of publications within a certain research field (Garfield, 1987; Rahaman et al., 2021). CiteSpace, which is a java-based information visualization software, was developed by Dr. Chaomei Chen (School of Information Science and Technology, Drexel University, Philadelphia, PA, United States) (Chen, 2004). This software has been widely used for bibliometric analysis and knowledge visualization of scientific literature data. This software allows knowledge network maps, such as cooperation network maps, co-citation network maps, and co-occurrence network maps, to be built. Using these maps, information is readily accessible for readers to intuitively understand the research hotspots and development processes of various knowledge domains (Zhou et al., 2022). In medical research, CiteSpace-based bibliometric analysis has been extensively conducted to allow scientists to explore the structures and dynamics of a certain field during a given period (Maggio et al., 2022), so as to establish its general outline and discovering future research directions (Janmaijaya et al., 2018).

Hitherto, to the best of our knowledge, there has been no published bibliometric analysis work in the field of TCE regarding neurodegenerative diseases. Therefore, in this study, we conducted a bibliometric analysis based on CiteSpace to systematically analyze research themes, hotspots, and new frontiers of the application of TCE in neurodegenerative diseases, offering a valuable approach besides traditional systematic reviews to understand the current research and emerging trends in this field.

## Materials and Methods

### Data Collection and Search Strategy

All data were collected from the Web of Science Core Collection (WoSCC). The three indexes, namely, the Science Citation Index Expanded (SCI-Expanded), the Social Sciences Citation Index (SSCI), and the Emerging Sources Citation Index (ESCI), were selected from the WoSCC as the data source. The search was performed on 17 March 2022. All original research articles and reviews in English were retrieved. The time span was from inception to 31 December 2021. To ensure the breadth of the search scope, we included as many search terms related to Traditional Chinese Exercises or neurodegenerative diseases as possible (Table 1). A total of 220 papers were collected and exported in plain text format with full records and references.

**TABLE 1:** Summary of literature selection in this study.

Content			
Data source	Web of Science Core Collection		
Time span	Inception-2021		
Languages	English		
Literature types	Article or review		
Search strategy	#1	4099	[TS = (tai-ji OR “Tai Chi” OR “Chi, Tai” OR “Tai Ji Quan” OR “Ji Quan, Tai” OR “Quan, Tai Ji” OR “Taiji” OR “Taijiquan” OR “T’ai Chi” OR “Tai Chi Chuan” OR qigong OR “qi gong” OR “chi gong” OR “ch’i Kung” OR “baduanjin” OR “ba duan jin” OR “wuqinxi” OR “yijinjing” OR “yi jinjing” OR “liuzijue” OR “traditional exercise” OR “Chinese traditional exercise” OR “traditional Chinese exercise” OR “Chinese exercise” OR “mind-body exercise”)]
	#2	473263	[TS = (“multiple sclerosis” OR “amyotrophic lateral sclerosis” OR “Parkinson’s” OR “Parkinson disease” OR “Alzheimer’s” OR “Alzheimer disease” OR “Huntington’s” OR “Huntington disease” OR Neurodegenerative)]
	#3	220	#1 AND #2 AND [DT = (“ARTICLE” OR “REVIEW”) AND LA = (“ENGLISH”)]

### Analysis Tool

All valid data were imported to Microsoft Excel 2019 and CiteSpace (5.8R3) for performing visual analysis. Microsoft Office Excel 2019 was utilized to analyze the trend of the number of articles published by year. CiteSpace was used to visually analyze countries/regions, institutions, authors, journals, cited references, keywords, as well as keywords with strong citation bursts over time. Related CiteSpace visualization knowledge maps, which consist of nodes and links, were drawn. The links represent the cooperation, co-occurrence, and co-citation relationships between two nodes. The nodes represent countries, institutions, authors, journals, and cited references. Nodes with a larger size indicate higher occurrence or citation frequency (Chen et al., 2014). The color of nodes indicates the occurrence or citation years. Centrality is an index for quantitatively evaluating the importance of a node in a network, and a centrality greater than 0.1 is considered significant. The outer purple trim of a node indicates high centrality, and nodes with high centrality are regarded as pivotal points or key points in a specific field (Liang et al., 2018).

## Results

### Publication Years

**FIGURE 1:**
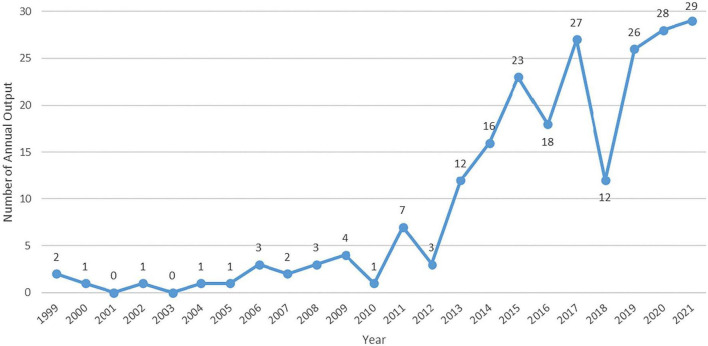
The annual number of publications on TCE for neurodegenerative diseases between 1999 and 2021.

A total of 220 papers, including 119 articles and 101 reviews, were obtained, excluding duplicates. As shown in Figure 1, the number trend of annual publications related to TCE combined with neurodegenerative diseases showed less growth before 2011, with less than 5 papers published per year on the topic. But this has shown a rapid growth from 2012 to 2015, a fluctuating growth from 2015 to 2021, and was steady at 26 or more papers per year in the last 3 years. These results indicate that this topic has received much more attention in the past decade than previously.

### Analysis of Countries/Regions and Institutions

**FIGURE 2:**
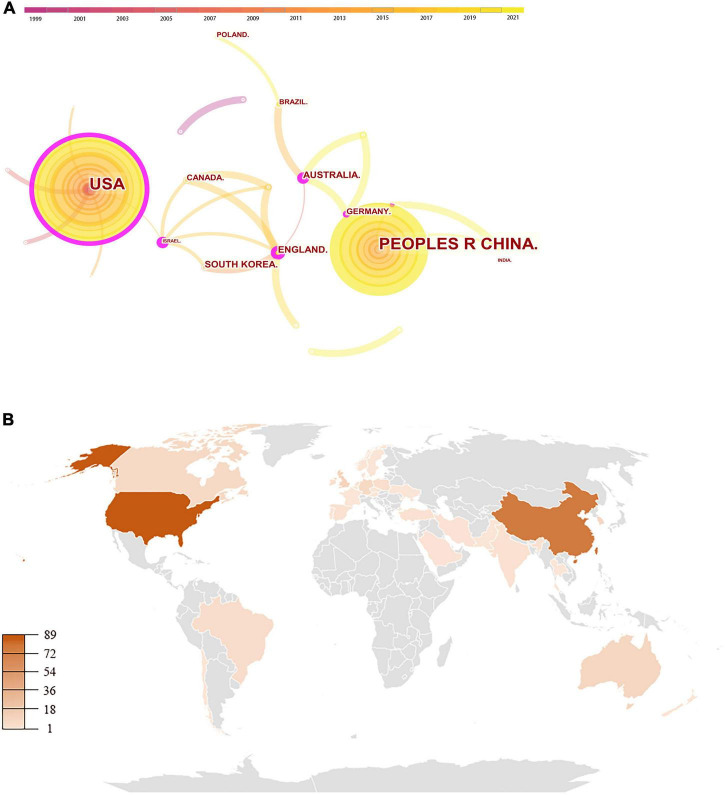
Visualization of countries/regions. **(A)** Collaboration network of countries/regions. The nodes in the map represent countries. The lines between the nodes represent cooperation relationships. The size of the node area shows the number of co-citations. The colors in the nodes represent the years. The purple ring represents centrality. **(B)** World map of publications distributed in various countries/regions.

**FIGURE 3:**
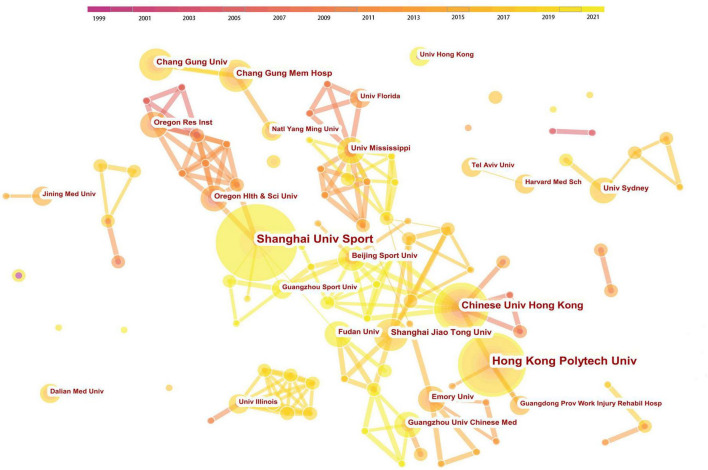
Collaboration network of institutions. The nodes in the map represent institutions, and lines between the nodes represent collaborative relationships. The size of the node area shows the number of co-citations. The colors in the nodes represent the years. The purple ring represents centrality.

As shown in Figure 2A, the country distribution map consists of 37 nodes and 28 links. The top 5 countries/regions with the highest number of papers were the United States, China, England, Australia, and South Korea, while the United States, England, Israel, and Australia were the only four countries/regions with a centrality value higher than 0.10 (Table 2). A detailed overview is presented as a world map (Figure 2B). The United States was the most dominant country in terms of both publication volume and centrality, holding 89 publications and a centrality value of 0.33. Although China came in second in the publication volume with 68 papers, its centrality was merely 0.08, indicating that the global influence of these papers was not proportional to their quantity. The co-institution network map is shown in Figure 3, with 262 nodes and 314 links. The top 3 prolific institutions were the Shanghai University of Sport, the Hong Kong Polytechnic University, and the Chinese University of Hong Kong, all of which are in China (Table 3). In terms of centrality, the Shanghai University of Sport had the highest centrality value of 0.09. These results illustrate that the United States was the most active and influential research country, while China had the most productive and influential institutes.

**TABLE 2:** Top 10 countries/regions in terms of publications and centrality.

Rank	Country/Region	Count (%)	Rank	Country/Region	Centrality
1	United States	89 (40.5%)	1	United States	0.33
2	China	68 (30.9%)	2	England	0.25
3	England	11 (5.0%)	3	Israel	0.25
4	Australia	10 (4.5%)	4	Australia	0.19
5	South Korea	9 (4.1%)	5	China	0.08
6	India	9 (4.1%)	6	Brazil	0.06
7	Germany	8 (3.6%)	7	Germany	0.02
8	Canada	8 (3.6%)	8	Netherlands	0.02
9	Brazil	6 (2.7%)	9	South Korea	0
10	Poland	5 (2.3%)	10	India	0

**TABLE 3:** Top 10 institutions in terms of publications and centrality.

Rank	Institution	Count (%)	Rank	Institution	Centrality
1	Shanghai univ sport	12 (5.5%)	1	Shanghai univ sport	0.09
2	Hong kong polytech univ	10 (4.5%)	2	Fudan univ	0.07
3	Chinese univ hong kong	8 (3.6%)	3	Shanghai jiao tong univ	0.06
4	Shanghai jiao tong univ	5 (2.3%)	4	Indiana univ	0.05
5	Chang gung univ	5 (2.3%)	5	Univ mississippi	0.04
6	Chang gung mem hosp	5 (2.3%)	6	Beijing sport univ	0.04
7	Univ mississippi	4 (1.8%)	7	Guangzhou sport univ	0.04
8	Oregon res inst	4 (1.8%)	8	Chinese univ hong kong	0.02
9	Emory univ	4 (1.8%)	9	Georgia inst technol	0.02
10	Fudan univ	4 (1.8%)	10	Delaware state univ	0.02

### Analysis of Authors

**FIGURE 4:**
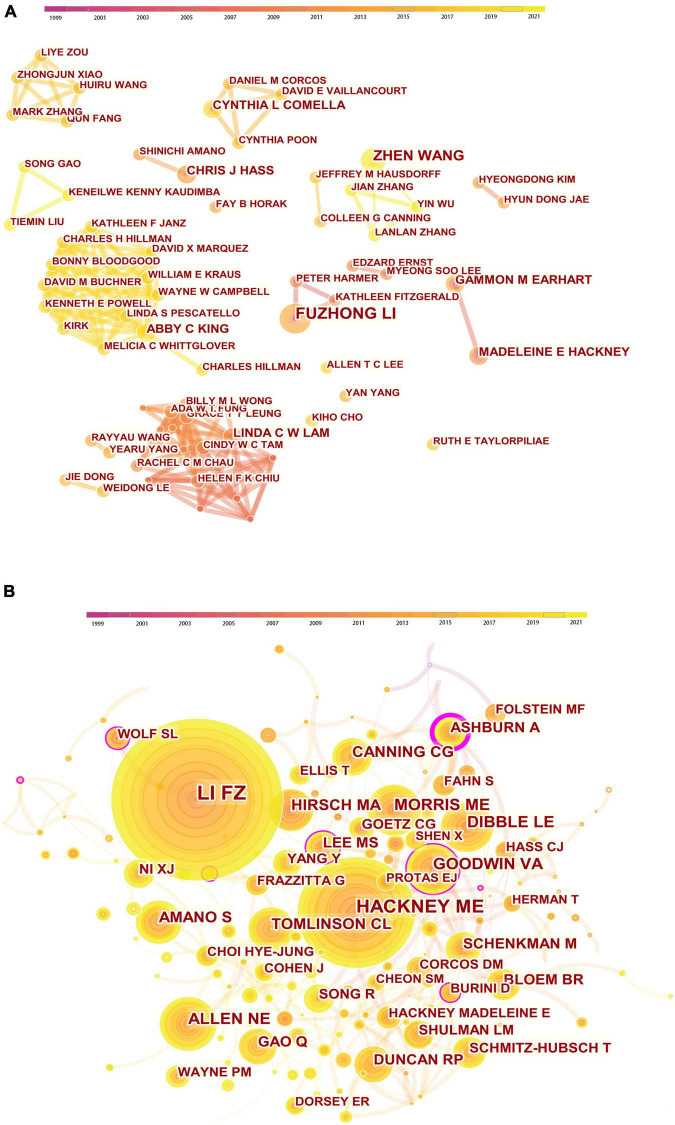
Visual analysis of authors. **(A)** Collaboration network of co-authors. The nodes in the map represent authors, and lines between the nodes represent the collaborative relationships. The colors in the nodes represent the years. **(B)** Network visualization map of cited authors. The nodes in the map represent co-cited authors, and lines between the nodes represent co-citation relationships. The colors in the nodes represent the years. The purple ring represents centrality.

The co-authorship and cited authors were analyzed to identify potential partnerships. The co-authorship network (Figure 4A) was composed of 350 nodes and 563 links. Among the authors, Fuzhong Li from the United States was the author who had the highest number of papers (*n* = 5), and this was followed by Zhen Wang (*n* = 4) from China (Table 4). All the other authors had only three or fewer papers each. It is worth noting that none of the authors had a significant centrality, indicating the lack of cooperation among these authors. Figure 4B displays the network of cited authors, with 622 nodes and 1,787 links. Fuzhong Li from the United States had the highest citation counts (*n* = 120), followed by Madeleine E Hackney (*n* = 85) from the United States and Natalie E Allen (*n* = 46) from Australia (Table 4).

**TABLE 4:** The top 5 prolific authors and cited authors.

Rank	Author	Country	Count	Centrality	Rank	Cited Author	Country	Count	Centrality
1	Li FZ	United states	5	0.06	1	Li FZ	United states	120	0.06
2	Wang Z	China	4	0.01	2	Hackney ME	United states	85	0.07
3	Earhart GM	United states	3	0.02	3	Allen NE	Australia	46	0.06
4	Hackney ME	United states	3	0.01	4	Morris ME	Australia	45	0.06
5	Lam L	China	3	0.01	5	Dibble LE	United states	43	0.02

### Analysis of Journals

The 220 papers were published in 136 journals. The 10 most productive journals published 59 papers, accounting for 26.8% of the total number of papers identified (Table 5). *PLOS ONE* was the most productive journal with 10 papers published, which was followed by *Evidence-Based Complementary and Alternative Medicine* with 8 publications. Among the 10 journals, *Movement Disorders* was the journal with the highest impact factor (IF), with an IF of 10.338. Besides, the analysis of journal co-citation was also performed to uncover the interdependence and cross-relationship between journals. The top-ranked journal by citation counts was *Movement Disorders* with 140 citations, followed by *Archives of Physical Medicine and Rehabilitation* (138 citations) and *Neurology* (129 citations). Among the 10 top-cited journals, *New England Journal of Medicine* had the highest IF of 91.253.

**TABLE 5:** The top 10 prolific journals and cited journals.

Rank	Journal	Count (%)	IF (2020)	Cited journal	Frequency	IF (2020)
1	PLOS ONE	10 (4.5%)	3.24	Movement disorders	140	10.338
2	Evidence-based complementary and alternative medicine	8 (3.6%)	2.63	Archives of physical medicine and rehabilitation	138	3.966
3	Neurorehabilitation and neural repair	7 (3.2%)	3.919	Neurology	129	9.91
4	Complementary therapies in medicine	5 (2.3%)	2.446	Parkinsonism and related disorders	125	4.891
5	Disability and rehabilitation	5 (2.3%)	3.033	New england journal of medicine	110	91.253
6	International journal of environmental research and public health	5 (2.3%)	3.39	Physical therapy	103	3.021
7	Journal of alternative and complementary medicine	5 (2.3%)	2.582	Journal of neurology neurosurgery and psychiatry	98	10.283
8	Movement disorders	5 (2.3%)	10.338	Journal of the american geriatrics society	97	5.562
9	Parkinsonism and related disorders	5 (2.3%)	4.891	Clinical rehabilitation	95	3.477
10	Archives of physical medicine and rehabilitation	4 (1.9%)	3.966	PLOS ONE	95	3.24

### Analysis of References

Table 6 demonstrates the top 5 most co-cited references. These references could be considered as the most popular papers in this field. The top co-cited reference was published in *New England Journal of Medicine*, which is the leading journal in clinical medicine, and was authored by Fuzhong Li et al. This study revealed that Tai Chi improved balance impairments and reduced falls in patients with PD. The second co-cited reference was published by Qiang Gao et al., which also found that Tai Chi exercise could improve the balance and decrease the fall risks in patients with PD. The third co-cited reference was published by Shinichi Amano et al., which suggested the use of short-term Tai Chi exercise for persons with PD should not be considered an effective means to improve dynamic postural control during gait initiation and gait performance.

**TABLE 6:** The top 5 cited references.

Rank	Title	Cited Frequency	Year	First Author	Journal	IF (2020)
1	Tai chi and postural stability in patients with parkinson’s disease	57	2012	Fuzhong Li	New england journal of medicine	91.253
2	Effects of tai chi on balance and fall prevention in parkinson’s disease: a randomized controlled trial	25	2014	Qiang Gao	Clinical rehabilitation	3.477
3	The effect of tai chi exercise on gait initiation and gait performance in persons with parkinson’s disease	23	2013	Shinichi Amano	Parkinsonism and related disorders	4.891
4	The impact of tai chi and qigong mind-body exercises on motor and non-motor function and quality of life in parkinson’s disease: a systematic review and meta-analysis	19	2017	Rhayun Song	Parkinsonism and related disorders	4.891
5	A randomized controlled trial of patient-reported outcomes with tai chi exercise in parkinson’s disease	18	2014	Fuzhong Li	Movement disorders	10.338

### Analysis of Keywords

**FIGURE 5:**
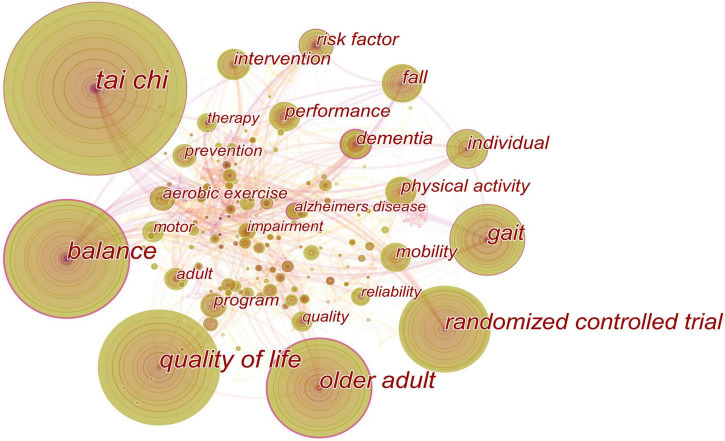
Co-occurring keywords map. The nodes in the map represent keywords. The lines between the nodes represent co-occurrence relationships. The purple ring represents centrality.

The keywords with a high frequency represent hot topics, while the keywords with a high centrality reflect the influence of corresponding research content in a research field. The keywords co-occurrence is illustrated in Figure 5, which consists of 366 nodes and 1,086 links. Table 7 demonstrates that the top 10 high-frequency keywords on this topic were Tai Chi, Parkinson’s disease, quality of life, balance, older adult, randomized controlled trial, gait, physical activity, individual, and fall. The top 10 high-centrality keywords were Alzheimer’s disease, balance, dementia, risk factor, aerobic exercise, gait, Tai Chi, cognitive function, basal ganglia, and older adult.

**TABLE 7:** The top 10 keywords in terms of frequency and centrality.

Rank	Count	Keywords	Rank	Centrality	Keywords
1	132	Tai Chi	1	0.36	Alzheimer’s disease
2	91	Parkinson’s disease	2	0.32	Balance
3	87	Quality of life	3	0.28	Dementia
4	66	Balance	4	0.22	Risk factor
5	59	Older adult	5	0.21	Aerobic exercise
6	51	Randomized controlled trial	6	0.18	Gait
7	26	Gait	7	0.16	Tai Chi
8	25	Physical activity	8	0.14	Cognitive function
9	21	Individual	9	0.13	Basal ganglia
10	20	Fall	10	0.12	Older adult

**FIGURE 6:**
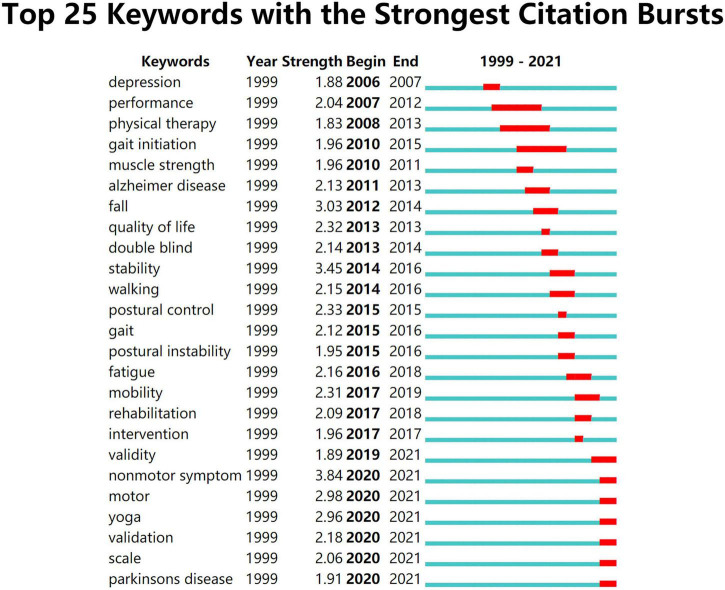
Top 25 keywords with the strongest citation bursts. The timeline is depicted as a blue line, and the time interval that a subject was found to have a burst is shown as a red segment, which indicated the beginning year, the ending year, and the duration of the burst.

A citation burst refers to the increasing frequency in citation within a certain time interval, which can reflect the development of cutting-edge research topics (Chen et al., 2012). The burst detection in CiteSpace is based on Kleinberg’s algorithm (Kleinberg, 2003). Figure 6 shows the top 25 keywords with the strongest citation burst from 1999 to 2021. The red line indicates the time period of the keyword burst, while the blue line represents the time interval. The keyword “non-motor symptom,” which appeared in 2020, was the keyword with the strongest citation bursts. There were seven burst keywords (i.e., validity, non-motor symptom, motor, yoga, validation, scale, and Parkinson’s disease), which continued to 2021.

## Discussion

### General Information

In this study, a total of 220 publications were acquired from the Web of Science Core Collection, from inception to 31 December 2021, most of which were published after 2012. The relatively small number of studies before 2010 suggests that the research was still in its infancy. The significant overall growth in the year of 2010 indicated the increasing attention in the international arena on TCE for treating neurodegenerative diseases. Another bibliometric study of TCE regarding improving cognitive function (Li W. et al., 2021) also found a significant growth trend in annual publications since 2012, indicating that TCE has indeed experienced a period of rapid development in the last decade. The major growth in publications regarding TCE provided strong support for TCE to expand out of China and go international. From 2019 to 2021, the annual number of papers increased steadily and slightly. It can be inferred that there will still be a relatively large number of publications in this field published in the coming years.

Analyzing the literature sources, 37 countries/regions published 220 papers. Many Western developed countries, such as the United States, England, Australia, and Germany, ranked high in the number of papers. This reflects the popularity and wide interest of TCE in the management of neurodegenerative diseases in the Western world. The United States was the most influential country in this field, which ranked first in terms of the publication volume and centrality. This is likely due to the fact that the United States is the world’s largest economy. Moreover, some outstanding experts in this field, such as Fuzhong Li, were based in the United States. Although TCE originated and flourished in China, in this study, China held a non-significant centrality with fewer papers than the United States. This may be related to the fact that many Chinese scholars like to publish non-English articles. Besides, there were 37 countries/regions and 28 links in the cooperation network of countries/regions (Figure 2A), indicating the lack of international communication. Therefore, more attention should be paid to the cooperation between countries to improve the development of this field. The analysis of institutions showed that some institutions in China, led by Shanghai University of Sport, had taken the lead in global research on this topic. These sterling research institutions are the key in improving the academic influence of China in this field.

From the perspective of authors, Fuzhong Li from the Oregon Research Institute in the United States was both the most prolific author and the most cited author. Li has been engaged in examining the effects of exercise, including Tai Chi, on balance, physical functioning, and the risks of falls for over two decades. The top 5 prolific authors and the top 5 cited authors are mainly from the United States, indicating the strong academic influence and great contributions of American researchers in this field. Through a network of co-authors, the cooperative relationships between scholars are distributed in a decentralized manner, suggesting that most authors prefer to cooperate with authors in a certain circle, especially from the same institution. This may partly explain why the topmost prolific authors had a non-significant centrality. Therefore, strengthening cooperation among authors from different institutions or countries should be encouraged.

In the analysis of journals, the most prolific journal was *PLOS ONE*, while the most cited journal was *Movement Disorders*. Among the top 10 published journals, only the journal *Movement Disorders* held an impact factor (IF) greater than 10.000, while the others had an IF between 2.446 and 4.891. This shows the difficulty in publishing high-impact papers in this field. The top 10 cited journals, however, generally had a greater IF, with the highest IF being 91.253 for *New England Journal of Medicine*. These top cited journals, to a certain extent, reflect the best source in the research field of TCE for neurodegenerative diseases.

According to the analysis of references, the top 5 cited references were all published between 2012 and 2017. All of these five papers had explored Tai Chi or Qigong as an intervention for PD, and four of them were clinical studies while the remaining one was a meta-based systematic review. Their widespread citation indicates that they are influences in this field. It is noteworthy that Fuzhong Li, who was also the most prolific and most cited author, held two of the top 5 cited papers. This reflects his high academic influence and significant contributions in this research field.

### Hotspots in Traditional Chinese Exercises for Neurodegenerative Diseases

In this study, we used keyword frequency, keyword centrality, and keyword bursts as indicators to reveal the research hotspots of the field. We summarized these research hotspots as (a) Tai Chi, (b) PD, (c) AD, (d) older adults, and (e) falls reduction.

### Tai Chi

As a representative type of TCEs, Tai Chi is a form of mind-body exercise with mild-to-moderate intensity, which is already recommended as a regimen of life promotion in China and other countries (Lan et al., 2013). Tai Chi is characterized by slow, smooth, and graceful movements such as body rotation, weight shift, slow strides, and single-leg standing, which challenge postural stability and balance and could improve neuromuscular function (Kuramoto, 2006; Lan et al., 2013). Beneficial effects of Tai Chi on balance control, cognitive function, cognitive-motor coordination, and limb muscle strength in patients affected by neurodegenerative diseases have been well reported, and Tai Chi intervention is more effective when combined with medications (Hong et al., 2000; Lam et al., 2012; Wu et al., 2013; Song et al., 2014). However, controversy still exists on the “dose” of Tai Chi intervention, which includes training methods, frequencies, and durations. The clinical studies in the field largely had small sample sizes, short observation time, and diversity regarding study design and styles of Tai Chi, which made them fail to fully reflect the therapeutic effects of Tai Chi (Gow et al., 2017; Yu et al., 2021). Hence, further randomized controlled clinical trials with a larger sample size and adequate intervention time are still needed to confirm the effects of Tai Chi for neurodegenerative diseases.

### Parkinson’s Disease

As a kind of movement disorder characterized by basal ganglia degeneration, PD mainly affects middle-aged and elderly adults. Exercise has been considered as a key component in the management of PD and might significantly improve the self-care ability and quality of life in patients with PD (Kedzior and Kaplan, 2019; Klein et al., 2019; Chen et al., 2020). Research has demonstrated that TCE, as complementary and alternative medicine (CAM) therapies, are able to improve motor and non-motor functions in patients with early to middle stage PD (Carvalho et al., 2021; Fogaca et al., 2021; Shen et al., 2021; Wan et al., 2021). For patients with later-stage PD, however, physical exercise intervention might be hard to complete due to the severe rigidity and poor condition of patients.

### Alzheimer’s Disease

Alzheimer’s disease is the predominant form of dementia that mainly occurs in the aged population (Anderson, 2019). The common symptoms of AD include progressive cognitive dysfunction, memory loss, and amnesia. Patients with AD often lose the ability to take care themselves, which imposes a great burden on families, communities, and society. Although no effective treatments can stop or reverse the progression of AD currently, early detection, accurate diagnosis, and early intervention, including TCE are attractive strategies to slow the progression and reduce the burden on public health.

### Older Adults

In clinical research on TCE for neurodegenerative disease, study participants were mostly aged 60 years or older. This can be explained by the high prevalence of neurodegenerative diseases among the middle-aged and elderly population and the fact that retired people have more free time to focus on their health than younger ones. As the elderly population continues to grow in developed countries and China, the research on the intervention of age-related chronic diseases has become a hot topic of scientific and technological innovation.

### Falls Reduction

Falls are common and even life threatening for patients with neurodegenerative disease. Since most neurodegenerative diseases are progressive and currently have no cure, as the disease worsens and the patients get older, they are prone to have decreased body coordination, slower postural responses, and cognitive decline, which are associated with increased falls risk (Segev-Jacubovski et al., 2011). A previous meta-analysis-based study found that exercise interventions which target balance, gait, and muscle strength increase, such as Tai Chi, can effectively reduce fall rates in older adults (Sherrington et al., 2020).

### Future Research Directions

The most popular keywords varied over the years changing from depression, performance, physical therapy to fall, stability, and finally to validity, non-motor symptom (Figure 6). Most of the burst keywords are related to PD, indicating that the research on TCE for PD has been a major trend in the past two decades. Besides, there has been much focus on clinical studies and systematic reviews, but less on basic studies or mechanism research. Based on the high-frequency and high-centrality keywords and the evolution of burst keywords, future research directions in this field could be concluded as follows:

(1) Parkinson’s disease is one of the most common neurodegenerative diseases, and patients with PD often manifest “motor” abnormalities like tremors, rigidity, “postural instability,” bradykinesia, and freezing, while non-motor symptoms including “fatigue” and “depression” are also prevalent among them. Many clinical trials and meta-analysis reporting TCE used to treat PD have been published in recent years (Cheon et al., 2013; Ni et al., 2014; Li Y. et al., 2021). PD will continue to be a focus of future research.
(2) To date, in the field, an overwhelming majority of research has focused on Tai Chi. Also, only a few neurodegenerative diseases have been researched, especially PD and AD. Future research is likely to explore other TCE, like Baduanjin, Wuqinxi, and Yijinjing. In addition, the effects of TCE in the management of other neurodegenerative diseases such as Huntington’s disease, multiple sclerosis, and amyotrophic lateral sclerosis should also be further studied.

This study still has some limitations. First, dual-map overlay and cluster analysis were not involved in this study. Second, we took the keywords as the topic of a paper to analyze, but some authors might not put important words that were present in the title as “keywords.” Thus, it might have the keyword analysis. Third, limitations of the CiteSpace software resulted in only the WoSCC database being searched for articles. Fourth, only articles and reviews were included excluding meeting records, letters, and books. Finally, only publications published in the English language were included; however, TCE likely receives research attention in non-English-speaking East Asian countries. Therefore, a more comprehensive bibliometric study which includes non-English papers and more databases such as China National Knowledge Infrastructure (CNKI) could be conducted in the future to present a broader overview of this field.

## Conclusion

This bibliometric analysis provides information on the most influential papers, hot spots of previous research, and future directions of the research on Traditional Chinese Exercise (TCE) for neurodegenerative diseases. The number of the publications in this field has shown major growth over the past decade. However, there is a great need for research institutions and authors to strengthen cooperation with researchers in other countries and institutions. Tai Chi, PD, Alzheimer’s dementia, older adults, and falls reduction are current research hotspots. Since the concentration of research is only in certain areas such as Tai Chi and PD, it is expected that other kinds of TCE, such as Baduanjin, Wuqinxi, and Yijinjing, may be studied in the future. In addition, the effects of TCE in the management of Huntington’s disease, multiple sclerosis, and amyotrophic lateral sclerosis have also yet to be studied. The findings from this bibliometric study may help researchers identify hot topics and discover new directions for future research in this field.

## Data Availability Statement

The original contributions presented in this study are included in the article/supplementary material, further inquiries can be directed to the corresponding author/s.

## Author Contributions

BJ drafted and revised the manuscript. CF drew all of the pictures. HH drafted the manuscript. DG reviewed and revised the manuscript. TH and ZL contributed to the conception and design of the study. All authors contributed to the article and approved the submitted version.

## Conflict of Interest

The authors declare that the research was conducted in the absence of any commercial or financial relationships that could be construed as a potential conflict of interest.

## Publisher’s Note

All claims expressed in this article are solely those of the authors and do not necessarily represent those of their affiliated organizations, or those of the publisher, the editors and the reviewers. Any product that may be evaluated in this article, or claim that may be made by its manufacturer, is not guaranteed or endorsed by the publisher.
